# Cardiomyocyte Protection by GATA-4 Gene Engineered Mesenchymal Stem Cells Is Partially Mediated by Translocation of miR-221 in Microvesicles

**DOI:** 10.1371/journal.pone.0073304

**Published:** 2013-08-28

**Authors:** Bin Yu, Min Gong, Yigang Wang, Ronald W. Millard, Zeeshan Pasha, Yueting Yang, Muhammad Ashraf, Meifeng Xu

**Affiliations:** 1 Department of Pathology and Laboratory Medicine, University of Cincinnati Medical Center, Cincinnati, Ohio, United States of America; 2 Department of Pharmacology and Cell Biophysics, University of Cincinnati Medical Center, Cincinnati, Ohio, United States of America; Northwestern University, United States of America

## Abstract

**Introduction:**

microRNAs (miRs), a novel class of small non-coding RNAs, are involved in cell proliferation, differentiation, development, and death. In this study, we found that miR-221 translocation by microvesicles (MVs) plays an important role in cardioprotection mediated by GATA-4 overexpressed mesenchymal stem cells (MSC).

**Methods and Results:**

Adult rat bone marrow MSC and neonatal rat ventricle cardiomyocytes (CM) were harvested as primary cultures. MSC were transduced with GATA-4 (MSC^GATA-4^) using the murine stem cell virus (pMSCV) retroviral expression system. Empty vector transfection was used as a control (MSC^Null^). The expression of miRs was assessed by real-time PCR and localized using *in situ* hybridization (ISH). MVs collected from MSC cultures were characterized by expression of CD9, CD63, and HSP70, and photographed with electron microscopy. Cardioprotection during hypoxia afforded by conditioned medium (CdM) from MSC cultures was evaluated by lactate dehydrogenase (LDH) release, MTS uptake by CM, and caspase 3/7 activity. Expression of miR-221/222 was significantly higher in MSC than in CM and miR-221 was upregulated in MSC^GATA-4^. MSC overexpression of miR-221 significantly enhanced cardioprotection by reducing the expression of p53 upregulated modulator of apoptosis (PUMA). Moreover, expression of PUMA was significantly decreased in CM co-cultured with MSC. MVs derived from MSC expressed high levels of miR-221, and were internalized quickly by CM as documented in images obtained from a Time-Lapse Imaging System.

**Conclusions:**

Our results demonstrate that cardioprotection by MSC^GATA-4^ may be regulated in part by a transfer of anti-apoptotic miRs contained within MVs.

## Introduction

Myocardial infarction (MI) is usually accompanied by cell death and loss of large numbers of cardiomyocytes (CM). Stem cells have the capacity to reverse acute and chronic injury in different experimental models and human clinical trials. Strategies incorporating the use of stem cells have emerged as promising approaches to support and enhance the intrinsic cardiac repair system [1], [2] by protecting native CM or increasing myocardial regeneration. Mesenchymal stem cells (MSC) obtained from bone marrow have been reported to improve cardiac function, reduce infarct size, and enhance myocardial regeneration following transplantation into infarcted myocardium [3]–[7]. Clinical studies using MSC have documented safety and feasibility in patients with acute MI and chronic ischemia [8]–[12]. It has also been reported that transplanted MSC participate in the myocardial tissue repair process by either transdifferentiation into CM and endothelial cells [4], [6], [13], [14], or by release of biologically active pro-angiogenic and cardioprotective factors [15]–[20]. Intramuscular or intracoronary injection of conditioned medium (CdM) or concentrated active factors secreted from MSC are therapeutically effective for treating heart failure [18]–[20] – suggesting that paracrine factors from MSC promote functional recovery of the infarcted heart. Indeed, most of the beneficial effects of stem-cell-based therapies have been attributed to soluble factors released from cells. Recent studies suggest that microvesicles (MVs) may play a relevant role in the intercellular communication between MSC and adjacent cells [21]–[23]. It has been reported that MVs not only contain soluble factors, but also miRs and mRNA.

miRs are very small noncoding post transcription regulating RNAs. miRs have been shown to exert a critical role in regulating mRNA in heart diseases and cell apoptosis. Overexpression of miR-221/222 induces cell survival whereas knockdown of these miRs is accompanied by increased apoptosis by dis-inhibiting p53 upregulated modulator of apoptosis (PUMA) [24], [25]. PUMA, a well-known pro-apoptotic member of the Bcl-2 protein family, acts by binding and inactivating anti-apoptotic Bcl-2 family members [26]. Upregulation of anti-apoptotic Bcl-2 family members (e.g., Bcl-2, or Bcl-w, or Bcl-x_L_) [27], [28], or the combined loss of pro-apoptotic Bcl-2 family members Bax and Bak [29] increases cells resistance to many apoptotic stimuli.

We transduced the GATA-4 gene into MSC and found that hearts transplanted with these MSC had an improved left ventricle (LV) function and a smaller infarct size than achieved with MSC controls. Moreover, we have demonstrated previously that MSC overexpressing GATA-4 release more growth factors and promote endothelial cell mediated angiogenesis [30]. GATA-4 can regulate miR expression in CM and increase CM resistance to ischemic injury [31]. In this study, we investigated whether miRs in MSC can be carried and translocated by MVs into extracellular spaces and affect the functions of neighboring cells. Specifically, we determined whether these miRs can regulate the expression of target proteins in CM and result in cardioprotection.

## Methods

### Ethics Statement

All animals were treated in accordance with the guidelines for the Care and Use of Laboratory Animals prepared by the National Academy of Sciences and published by the National Institutes of Health (NIH publication No. 85-23, Revised 1996). Studies were conducted according to a protocol approved by the Institutional Animal Care and Use Committee, University of Cincinnati.

### Primary MSC Culture

MSC were obtained from femurs and tibias of male Sprague-Dawley (SD) rats (2∼4 month) as described previously [5], [32]. In brief, femurs and tibias were removed from rat sacrificed with anesthesia overdose. Bone marrow cells were flushed and cultured with Iscove's modified Dulbecco's medium (IMDM) (Gibco) supplemented with 15% fetal bovine serum (FBS) and penicillin (100 U/mL)/streptomycin (100 µg/mL) at 37°C in humid air with 5% CO_2_. After being seeded for 2 days, MSC adhered to the bottom of culture plates, while hematopoietic cells remained in suspension. The culture medium was changed every 3 days.

The second passage of MSC was used to transduce recombinant GATA-4 based on our previous report [30]. Retrovirus expressing GATA-4 was constructed using a pMSCV retroviral expression system (Clontech). IRES-EGFP was cloned into pMSCV vectors at XhoI and EcoRI sites, and then GATA-4 was excised from pcDNA-GATA-4 and cloned into pMSCV-IRES-EGFP. GP2-293 cells (Clontech) were co-transfected with pMSCV-GATA-4-IRES-EGFP and pVSVG following the manufacturer's instruction. Vector empty control GP2-293 cells were also co-transfected with pMSCV-IRES-EGFP and pVSVG. After 48 hours, the supernatants were collected and filtered through 0.45 um syringe filter. MSC were incubated with these supernatants in the presence of 10 µg/ml polybrene (Sigma) for 12 hours. The transfection efficiency was about 100% as assessed by fluorescence microscopy for GFP and by immunostaining and quantitative real-time PCR of GATA-4 after treatment with puromycin (3 µg/ml) (Sigma) for 5 days.

### CM Culture and Characterization

CM were isolated from heart ventricles of neonatal rats (1 to 3 days old) as described previously [32]. Ventricles were minced into 1 mm^3^ pieces and digested with trypsin and collagenase II using a commercially available neonatal CM isolation kit (Worthington Biochemical Co.). The cells were re-suspended in Dulbecco's modified Eagle's medium (DMEM) supplemented with 10% FBS and penicillin (100 U/ml)/streptomycin (100 µg/ml). To enrich CM yield, dissociated cells were pre-plated for 2 hours to allow non-myocytes to attach to the bottom of the culture dish and be subsequently discarded.

Immunocytochemistry was performed to verify CM purity as described previously by our laboratory, with modifications [32]. Cells cultured on glass coverslips were fixed in 4% paraformaldehyde (PFA) and then incubated with the mouse monoclonal anti-sarcomeric α-actinin (Sigma) and rabbit polyclonal anti-connexin43 (Santa Cruz). After thorough washing, secondary antibodies of goat anti-mouse IgG (FITC-conjugated) and goat anti-rabbit IgG (Alex-546-conjugated) were applied. Nuclei were stained with 4′,6-diamino-2-phenylindole (DAPI). Fluorescent images were obtained using an Olympus BX 61 microscope equipped with a digital camera (Olympus).

### Preparation of Concentrated CdM and MVs

MSC were trypsinized and seeded at 4×10^6^ cells per 15-cm plate. After 24 hours, regular culture medium was replaced with 15 ml of serum- and antibiotic-free medium. Forty-eight hours later, medium was collected and centrifuged to remove cell debris. The supernatant was transferred to ultra-filtration conical tubes (Amicon Ultra-15), and centrifuged (3,000 g for 45 minutes at 4°C) to concentrate CdM to 100×.

MVs were isolated from CdM using ExoQuick-TC Exosome Precipitation Solution per manufacturer's instructions (System Biosciences) (SBI). Purified MVs were characterized by western blot using three biomarkers: CD9, CD63, and HSP70. The morphology of MVs was observed under transmission electron microscope (JEOL).

### MVs Internalization

Freshly isolated CM were plated in specialized Hi-Q4 culture dishes at a density of 2×10^4^/dish and cultured in DMEM containing 10% FBS for 24 hours. MVs (10 µg protein) were pre-labeled with PHK26 (Sigma) and added to CM cultures. Images were acquired under 20× magnification at 37°C and in humid air with 5% CO_2_. Time-lapse images were taken in both phase-contrast and red fluorescence channels from Biostation IM-Q System (Nikon).

### Real Time PCR for miRs Expression

Total RNA from cells and MVs was extracted using mirVana™ miR isolation kit (Ambion) following manufacturer's protocol. cDNA was synthesized using miScript Reverse Transcription Kit (QIAGEN). Quantitative PCR was performed with miR specific primers and miScript SYBR Green PCR Kit (QIAGEN) on iQ5 real-time PCR system (Bio-Rad). U6 snRNA was used as an internal control. Relative expression of miRs was calculated based on the threshold cycle with different efficiency of each primer.

### miR Transfection

Lentiviral expression system was used to perform effective overexpression of miR in MSC. Lenti-miR 221-copGFP (lenti-miR-221) construct and scramble-copGFP control (lenti-miR-NC) construct were directly purchased from SBI. 293TN cells were co-transfected with either the expression vector or control vector and pPACK H1 packaging plasmid using Pure Fection transfection reagent (SBI) according to manufacturer's instruction for production of high titer pseudo-lentiviral particles. The cultured medium containing packaged pseudo-viral particles was collected at 72 hours post-transfection and concentrated with PEG-it™ virus precipitation solution (SBI). The Global Ultra Rapid Lentiviral Titer Kit (SBI) was used to determine the titers of pseudo-viral particles. Finally, a 50–70% confluent second passage of MSC was infected with the generated viral particles at 5–10 multiple infection. Seventy-two hours later, 70–90% transduction efficiency was documented based on the amount of copGFP green fluorescence positive cells.

### In Situ Hybridization (ISH)

ISH was performed using a double-digoxigenin (DIG)-labeled miRCURY LNA miR detection probe against has-miR-221 (EXIQON) and IsHyb-ISH Kit according to manufacturer's instructions (BioChain). Cultured cells were fixed with 4% PFA at room temperature for 20 minutes and washed twice with 1× Phosphate Buffered Saline (PBS) for 5 minutes each. After treating with 4 µg/ml proteinase K (ROCHE) for 10 minutes at 37°C, the cells were washed once in 1× PBS for 5 minutes and fixed again with 4% PFA. Hybridization with the Locked Nucleic Acids (LNA) probe (10 nM) was carried out at 54°C for 16 hours after incubation in a pre-hybridization solution for 3 hours at 50°C. The LNA detection probe was heated in the hybridization solution at 80°C for 5 minutes to linearize it, then chilled on ice before applying it to the cells. The cells were washed with 2× saline-sodium citrate buffer (SSC) and 1.5× SSC once for 10 minutes at 54°C, respectively, then rinsed with 0.2×SSC twice (20 minutes each) at 37°C. Cells were incubated for 1 hour in blocking solution followed by 1∶200 PBS diluted alkaline phosphatase (AP)-conjugated anti-DIG antibody overnight at 4°C. After the cells were washed with PBS and AP buffer twice (10 minutes each), the slides were incubated with NBT/BCIP solution in the dark overnight.

### Luciferase Assay

Three separate algorithms (miRanda, TargetScan, and PicTar) were used to find potential target sites for miR. The miTarget™ dual luciferase reporter vector containing the full-length 3′ UTR sequence of PUMA (pEZX-MT01-PUMA) was obtained commercially (GeneCopoeia). To confirm the effect of miR-221 on 3′UTR of PUMA, transient co-transfection of lenti-miR-221 vector/or lenti-miR-NC and pEZX-MT01-PUMA in 293TN cells was performed. 293TN cells were seeded at a density of 5×10^4^/well in a 6-well plate 24 hours before transfection. Cells were then co-transfected using lipofectamine 2000 (Invitrogen) with 1 µg of lenti-miR-221, or with lenti-miR-NC and 1 µg of pEZX-MT01-PUMA. After 48 hours, cells were lysed and luciferase activities were measured using the Dual-Luciferase Reporter Assay System (Promega) on a luminometer (HIDEX, plate chameleon) according to the manufacturer's instructions (Promega). Results were normalized to renilla luciferase activity and data were expressed as relative luciferase activity.

### Electroimmunoblotting

The proteins were extracted from different cells or MVs using Qproteome Mammalian Protein Prep Kit (QIAGEN). Protein concentrations were quantified with DC protein assay reagent (Bio-Rad). Denatured proteins (30 µg) were then analyzed using 12% sodium dodecyl sulfate–polyacrylamide gel electrophoresis (SDS–PAGE). After electrophoresis, proteins were transferred to a polyvinylidene difluoride membrane. The membrane was blocked with 5% milk in Tris-buffer saline solution (pH 7.6) containing 0.05% Tween-20 (TBS/T), and then incubated, respectively, with following primary antibodies: polyclonal rabbit anti-PUMA (Cell Signaling), anti-HSP70 (Cell Signaling), anti-CD63 (Santa Cruz), anti-CD9 (Abcam), and anti-β-actin (Cell Signaling) overnight at 4°C. The membrane was then incubated for 1 hour with HRP conjugated goat anti-rabbit secondary antibody (Cell Signaling) at room temperature, washed and developed with the ECL plus kit (GE Healthcare). Densitometric analysis for the blots was performed with FluoChem SP software (Alpha Innotech).

### Cell Ischemic Injury

To mimic ischemic injury, 3-day CM cultures in serum-free low glucose DMEM were placed in a hypoxic incubator (Sanyo) at 37°C with 94% N_2_/5% CO_2_/1% O_2_ for 24 and 48 hours. CM injury was evaluated by lactate dehydrogenase (LDH), a stable cytosolic enzyme released upon cell lysis, measured with a commercially available kit (BioVision). Cell survival was determined by a novel tetrazolium compound, (3-(4,5-dimethylthiazol-2-yl)-5-(3-carboxymethoxyphenyl)-2-(4-sulfophenyl)-2H-tetrazolium, inner salt; MTS) assay with CellTiter 96® AQ_ueous_ One Solution Cell Proliferation Assay (Promega). Caspase-3/7 activity was used as an indicator of apoptosis (Caspase- Glo**®** 3/7 Assay Kit, Promega). The absorbance at 450 nm (for LDH), 490 nm (for MTS), and luminescence (for caspase 3/7) of each sample were read on a microplate M3 spectrophotometer (Molecular Devices).

### Statistical Analysis

All data were obtained in at least three independent experiments. Quantitative data are presented as the mean ±SEM. Data were analyzed using GraphPad Prism (GraphPad Software). Statistical comparisons were performed using unpaired two-tailed Student's *t* tests and one-way analysis of variance. The Holm-Sidak method and/or Bonferroni correction was used to determine the significance of differences in multiple comparisons. A value of *p*<0.05 was considered statistically significant.

## Results

### MSC derived CdM protects CM against hypoxic injury

**Figure 1:**
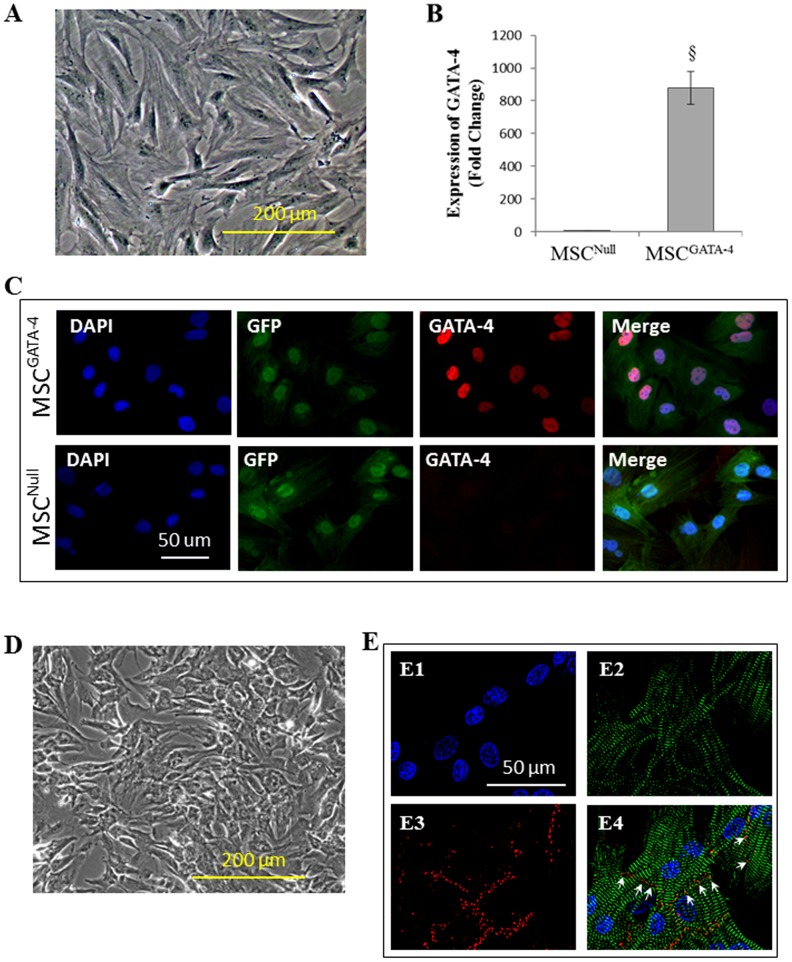
Morphology of cultured MSC and CM. Panel A: MSC obtained from rat bone marrow. Panel B: Quantitative real-time PCR of GATA-4 expression in MSC transduced with GATA-4 (MSC^GATA-4^) or empty vector (MSC^Null^). Panel C: Immunostaining of MSC^GATA-4^ and MSC^Null^. Panel D: CM obtained from rat neonatal ventricles. Panel E: CM were immunostained positive for α-actinin (green) and connexin 43 (red, white arrows). Myofibers were seen with clear Z-lines in sarcomeres. E1: DAPI; E2: α-actinin; E3: connexin 43; and E4: merged images of E1 to E3. ^§^, p<0.05 *vs* MSC^Null^.

MSC obtained from femurs and tibias appeared as a morphologically heterogeneous population. These cells exhibited fibroblast-like morphology with irregularly shaped large euchromatic and lobulated nuclei as seen in Figure 1A. MSC^GATA-4^ exhibited a higher expression of GATA-4 measured using quantitative real-time PCR (Figure 1B). Both MSC^GATA-4^ and its empty vector control (MSC^Null^) expressed GFP, only MSC^GATA-4^ stained intensely for GATA-4 (Figure 1C). CM displayed a flattened shape with cell clusters (Figure 1D). CM were significantly smaller than MSC and had spontaneous beating. CM were positive for α-actinin and expressed the gap junction protein connexin 43 (Figure 1E). Clear Z-lines in sarcomeres were observed in CM myofibers.

**Figure 2:**
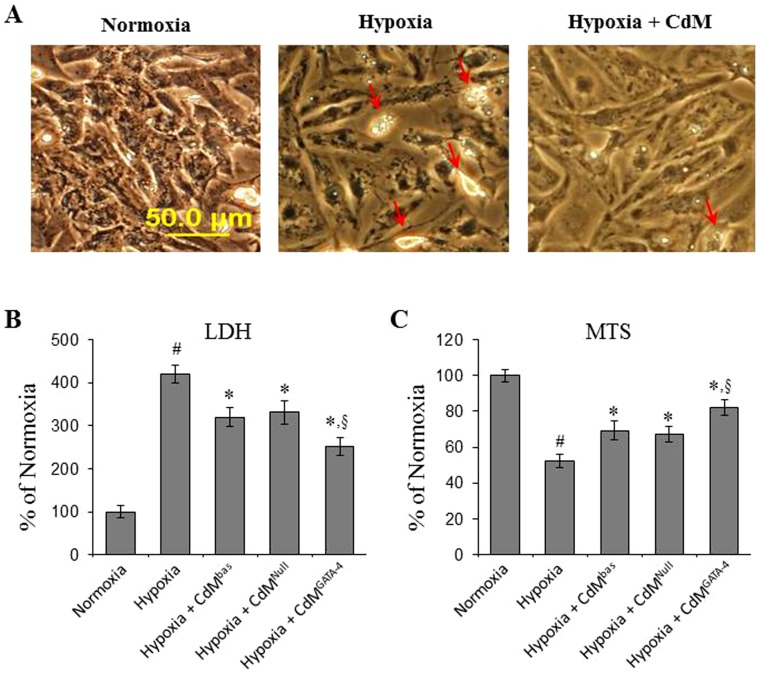
CdM derived from MSC cultures protects CM against ischemic injury induced by exposure to hypoxia for 48 hours. Panel A: CM morphology in different groups visualized under inverted microscopy. Panel B: LDH release from CM. Panel C: MTS uptake by CM. ^#^, p<0.05 *vs* normoxic control. *, p<0.05 *vs* hypoxic control; ^§^, p<0.05 *vs* CdM^Null^ treated CM. CdM^bas^: CdM derived from MSC^bas^; CdM^Null^: CdM derived from MSC^Null^; CdM^GATA-4^: CdM derived from MSC^GATA-4^.

After being exposed to hypoxia for 48 hours, CM exhibited sluggish beating. Cell density decreased with cells displaying a shrunken morphology and an increase in number of apoptotic cells compared to CM cultured in normoxia. LDH release from CM was significantly increased and cell uptake of MTS was reduced. However, the morphology of CM was significantly protected in CM treated with CdM. LDH release was significantly decreased, and MTS uptake was increased in hypoxic CM treated with CdM compared to the hypoxic control group. The treatment of CdM obtained from MSC^GATA-4^ (CdM^GATA-4^) showed a beneficial protection on CM compared to CdM obtained from MSC^Null^ (CdM^Null^) (Figure 2).

### Overexpression of miR-221 enhances MSC mediated cardioprotection

**Figure 3:**
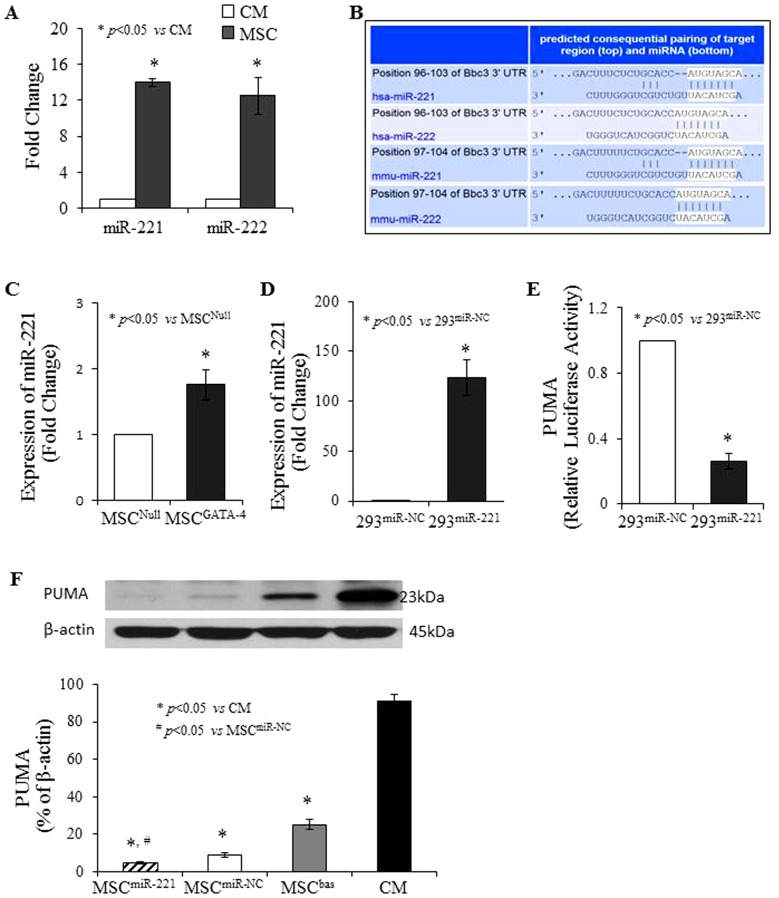
Expression of miR-221/222 and PUMA in MSC and CM. Panel A: miR-221/222 expression in MSC and CM assayed using real-time PCR. Panel B: TargetScan shows the 3′ UTR of PUMA containing the conserved miR-221/222 binding site. Panel C: miR-221 expression in MSC^GATA-4^ and MSC^Null^. Panel D: miR-221 expression in 293^miR-221^ and 293^miR-NC^. Panel E: PUMA in 293^miR-221^ and 293^miR-NC^, respectively. Panel F: Western blot of PUMA and corresponding semi-quantitative data in different cells. 293^miR-NC^ = 293TN transfected with lenti-miR-NC; 293^miR-221^ = 293TN transfected with lenti-miR-221; MSC^bas^ =  basal MSC; MSC^miR-221^ = MSC transfected with miR-221; MSC^miR-NC^ = MSC transfected with miR-NC.

The expression of miR-221/222 family in MSC was significantly higher than that in CM (Figure 3A). The 3′ UTR of PUMA [also recognized as Bcl-2 binding component 3 (Bbc3)] contains the conserved miR-221/222 binding sites (Figure 3B). Moreover, the expression of miR-221 in MSC^GATA-4^ was significantly higher than that in MSC^Null^ (Figure 3C). Transfection of miR-221 into 293TN cells resulted in a significant inhibition of the basal level of the PUMA related luciferase activity compared to the cell transfected with miR-NC (Figure 3D and 3E). Western blot analysis indicated that PUMA in MSC was significantly lower than that in CM. MSC transfected with miR-221 (MSC^miR-221^) significantly decreased PUMA protein levels compared with that in control MSC transfected with negative control miR (MSC^miR-NC^) (Figure 3F).

**Figure 4:**
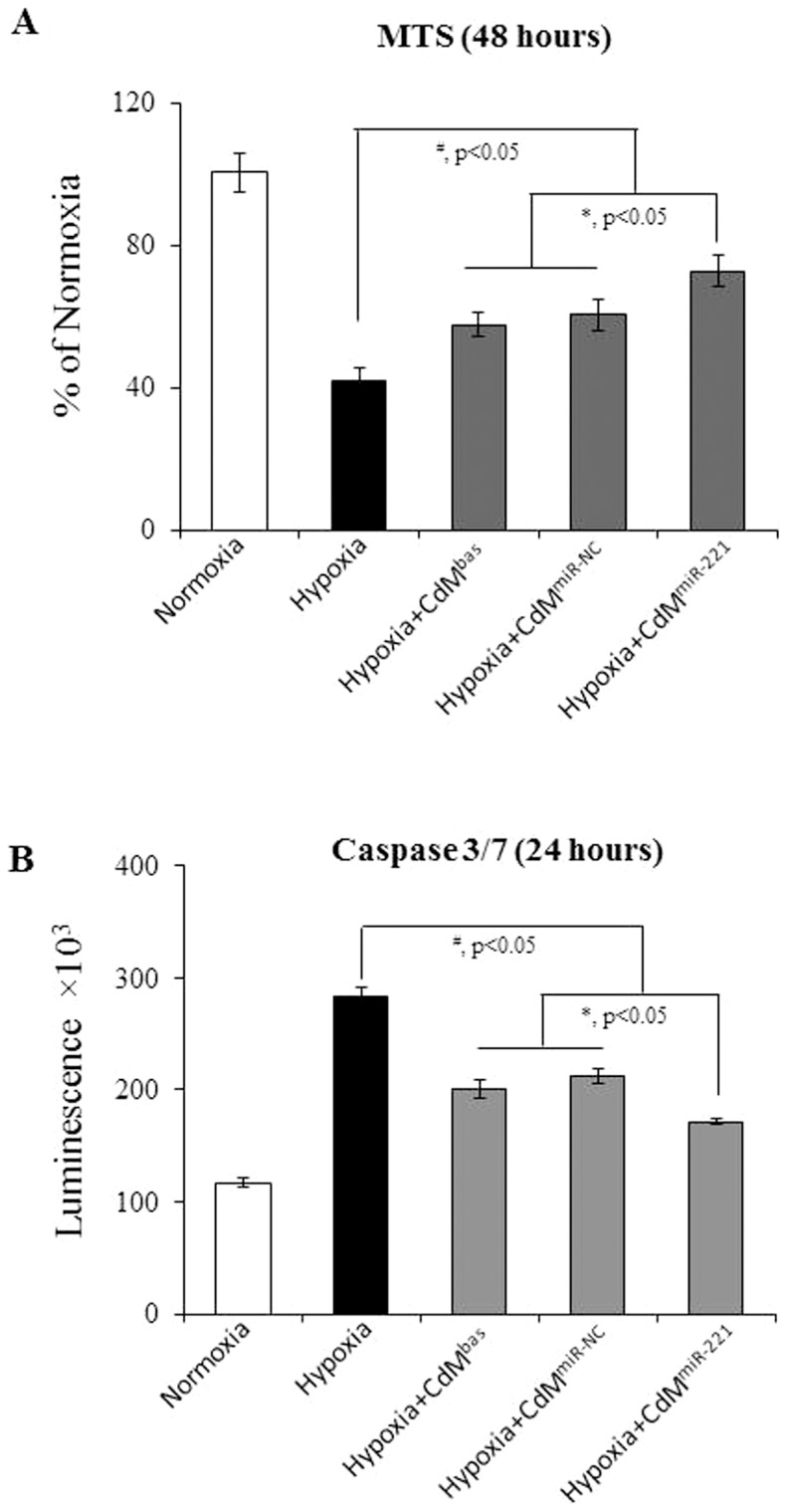
miR-221 expression enhances MSC-mediated cardioprotection against hypoxic injury. Panel A: MTS uptake by CM exposed to 48 hours hypoxia. Panel B: Caspase 3/7 activity in CM after 24 hours hypoxia. CdM^bas^: CdM derived from MSC^bas^; CdM^miR-NC^: CdM derived from MSC^miR-NC^; and CdM^miR-221^: CdM derived from MSC^miR-221^.

To evaluate whether miR-221 plays a role in MSC mediated cardioprotection, primary cultured CM were treated with CdM obtained from MSC^miR-221^ (CdM^miR-221^), MSC^miR-NC^ (CdM^miR-NC^), and basal MSC (CdM^bas^), respectively. MTS uptake in CM exposed to hypoxia for 48 hours was reduced to 40% compared to CM under normoxia, reflecting decreased CM survival (*p*<0.05 *vs* normal control). MTS uptake was significantly increased when CM were treated with CdM^bas^. Moreover, CM treated with CdM^miR-221^ increased MTS uptake to 73% of that in CM under normoxia, and significantly higher than that in CM treated with CdM^miR-NC^ (*p*<0.05) (Figure 4A). Apoptosis represents a crucial mechanism of CM loss in number of cardiac pathologies. Caspase-3/7 activity was significantly increased in CM exposed to hypoxia for 24 hours and was significantly reduced in CM treated with CdM^bas^. CdM^miR-221^ showed a more powerful potential in preventing caspase 3/7 activation induced by hypoxia comparing with CdM^miR-NC^ (Figure 4B).

### miRs transfer from MSC to CM – effect of miR-221 on PUMA levels

**Figure 5:**
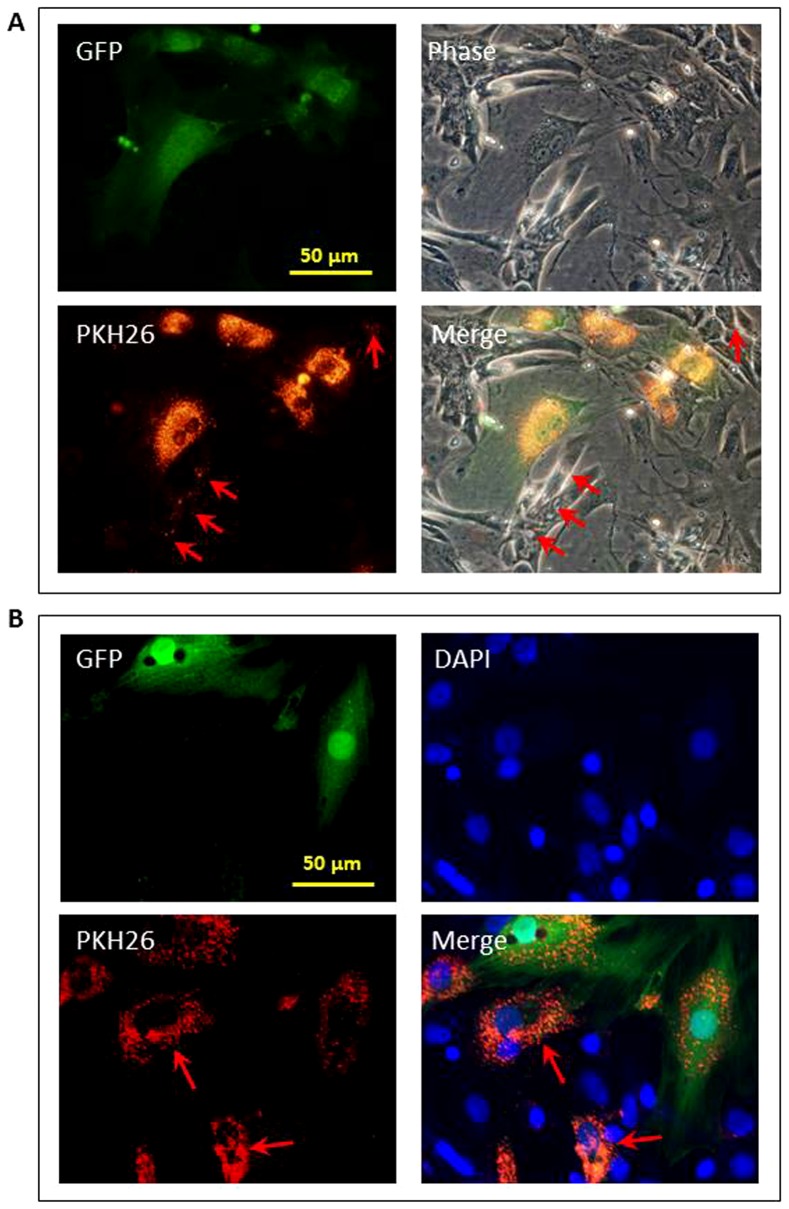
Transfer of bioactive molecules from MSC to CM. MSC pre-labeled with PKH26 were co-cultured with CM for 4 days. PKH26 was visible in GFP positive MSC and in some CM (red arrows). Panel A: co-culture of MSC and CM before fixation. Panel B: co-culture of MSC and CM after fixation and counterstained with DAPI.

**Figure 6:**
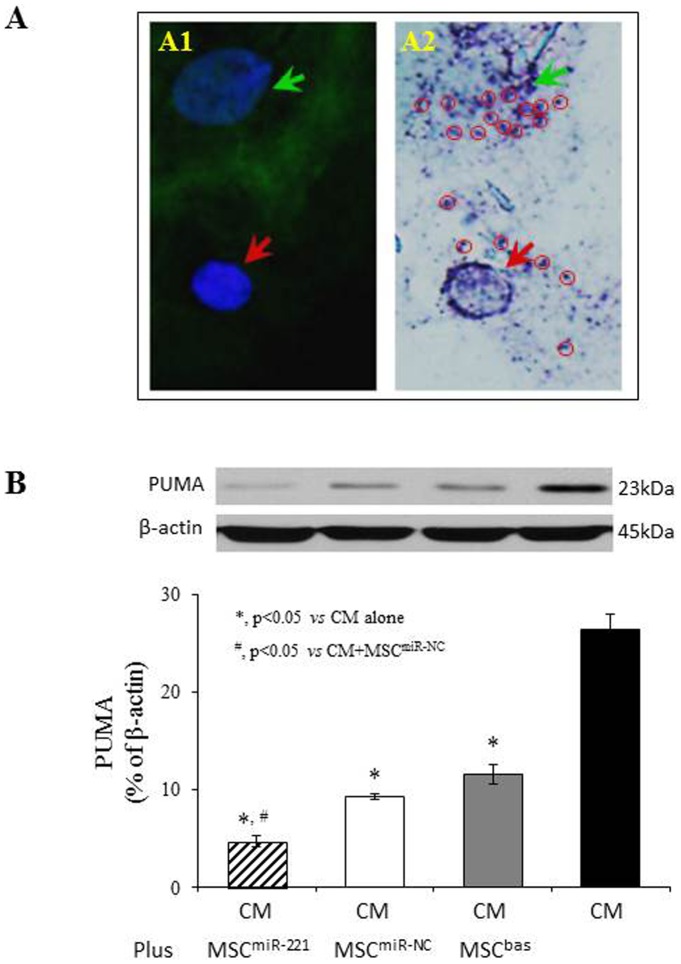
miR-221 transferred from MSC to CM and reduced PUMA expression in CM. Panel A: *In situ* hybridization staining of miR-221 in CM co-cultured with MSC^miR-221^ for 48 hours. miR-221 is shown as a purple-blue signal (red circles) which was observed in both GFP positive MSC (green arrow) and GFP negative CM (red arrow). Panel B: Western blot of PUMA and corresponding semi-quantitative data in CM co-cultured with various MSC in a dual-chamber system.

The transfer of bioactive molecules between MSC and CM was tracked using the red fluorescence of PKH26. MSC expressed green fluorescence following transfection with either lenti-miR-221 or lenti-miR-NC. MSC were pre-labeled with PKH26 and co-cultured with CM. PKH26 was not only visible in GFP positive MSC, but also in some CM after 4 days of co-culture with MSC (Figure 5A, red arrows). PKH26 appeared around nuclei of some GFP negative CM in cells fixed with 4% PFA and counterstained with DAPI (Figure 5B, red arrows). To directly detect the translocation of miRs from MSC to CM, ISH was performed after CM were co-cultured with MSC^miR-221^ for 48 hours. miR-221 location was detected as a purple-blue signal by incubating cell cultures with NBT/BCIP. miR-221 was observed in GFP positive MSC (green arrow) and in GFP negative cells (red arrow) (Figure 6A).

The effect of translocated miR-221 on the PUMA levels in CM was further investigated by co-culture CM with MSC in the dual chamber system. MSC were seeded in the upper chamber and CM in the lower chamber, respectively. Western blotting data showed that PUMA protein levels were markedly decreased in CM co-cultured with MSC^miR-221^ compared with CM cultured alone or CM co-cultured with MSC^miR-NC^ (Figure 6B).

### MVs mediate miRs translocation

**Figure 7:**
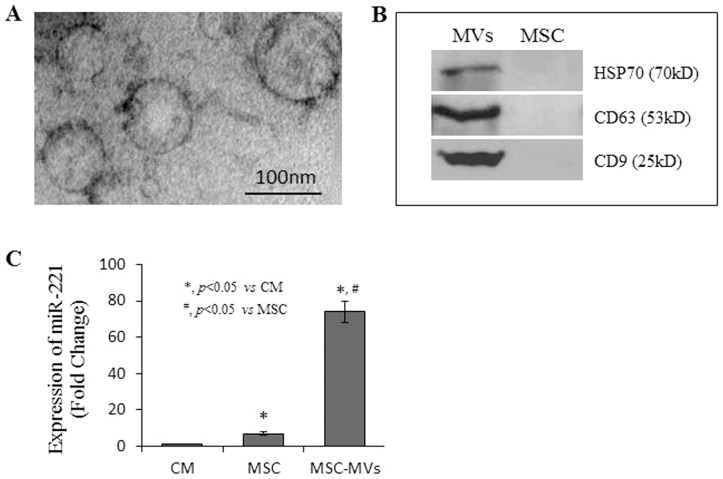
The morphology and characterization of MSC derived MVs (MSC-MVs). Panel A: Morphological features of MSC-MVs under electron microscope. Panel B: Western blot results show that MSC-MVs highly expressed HSP70, CD63, and CD9 compared to MSC; Panel C: The expression of miR-221 in MSC-MVs, MSC, and CM analyzed using real-time PCR.

MVs derived from MSC (MSC-MVs) exhibited a rounded morphology with a transparent center (Figure 7A). MSC-MVs highly expressed HSP70, CD63, and CD9, while the expression level of these proteins was lower in MSC (Figure 7B). Notably, the expression of miR-221 in MSC-MVs was significantly higher than that found in MSC (10.7-fold) or in CM (74.2-fold) (Figure 7C).

**Figure 8:**
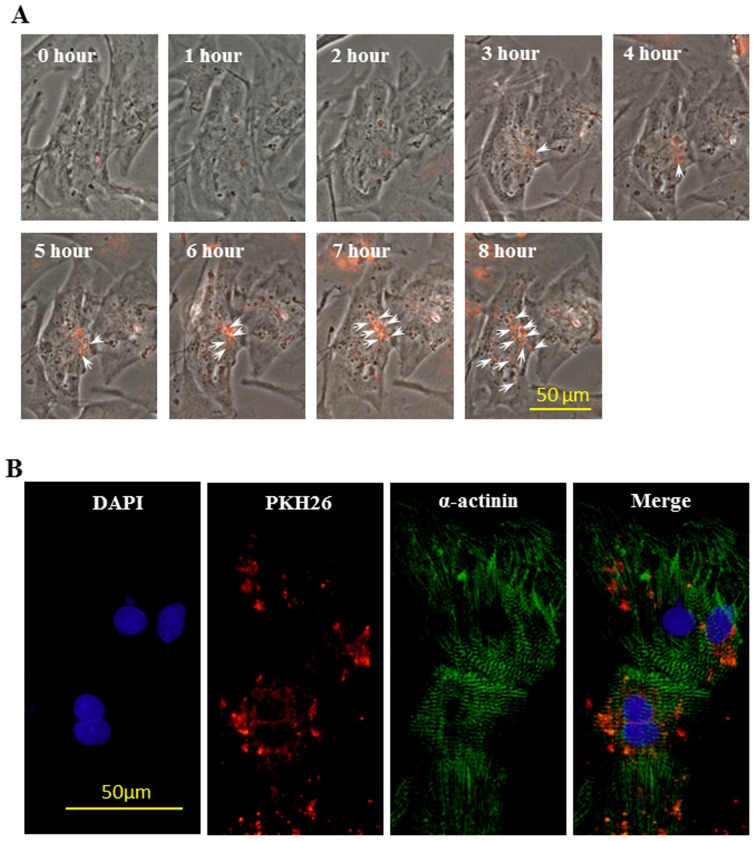
Internalization of PKH-26 pre-labeled MSC-MVs (MVs-PKH26) by CM. Panel A: Time-lapse images of MVs-PKH26 recorded at one hour intervals for 8 hours after addition of these MVs to CM culture. PKH26 red fluorescence (MVs) (white arrows) appeared inside CM at hour 3 and was widely scattered in whole cell clusters at hour 8. Panel B: Immunostaining shows internalization of MVs-PKH26 (red arrows) by α-actinin positive cells after MVs were added to CM cultures for 24 hours.

To study the internalization of MSC-MVs by CM, MVs pre-labeled with PKH26 (PKH-26-MVs) were added into 2×10^4^ cultured CM. One hour interval time-lapse images of CM for 8 hours following the addition of PKH26-MVs are shown in Figure 8A. No clear red MVs could be seen in CM during the first 2 hours. Very little but some red fluorescent spots appeared in CM by hour 3 (white arrows). PKH26-MVs were clearly visible in many CM by hour 8. To confirm whether MVs were transferred into CM, cultured cells were fixed and labeled with anti-α-actinin. Many PKH26-MVs were found inside α-actinin positive CM (Figure 8B).

## Discussion

In this study, we investigated that the mechanism by which MSC mediated cardioprotection is partially related to the translocation of miR from MSC to CM and to regulation of their target proteins in CM. We first investigated the cardioprotection by MSC-derived CdM. Then, we documented: 1) significantly higher expression of miR-221/222 family in MSC than in CM; 2) enhanced cardioprotection afforded by MSC^GATA-4^ and MSC^miR-221^; 3) reduction of PUMA expression in MSC by miR-221; 4) CM rapidly internalized MVs with high levels of miR-221 released from MSC; and 5) reduction of PUMA expression in CM by miR-221 transferred in MVs from MSC. Our studies indicate that MSC^GATA-4^ mediated cardioprotection is conferred, at least in part, by miR-221 carried in MVs. miR-221 down-regulates the expression of PUMA in CM.

Restoration of injured tissue is the primary goal of stem cell-based therapy for MI. In this study, we observed that CdM obtained from MSC protects CM against ischemic/hypoxic injury. Our results are consistent with the previous report by Angoulvant *et al*. [7]. Nguyen *et al*. demonstrated recently that a single intracoronary injection of concentrated biologically active factors secreted by MSC could achieve early protection of ischemic myocardium and improve cardiac repair and contractility [20]. In addition, CdM originating from MSC had been documented to contain higher concentrations of angiogenic (VEGF, IGF-1, endothelin, and epiregulin), anti-apoptotic (Galectin-3, Smad-5, sRFP-1, and sRFP-4) and anti-remodeling factors [5], [15], [20]. These factors play an important role in increasing CM resistance to ischemic injury and appear to improve regional perfusion of the ischemic heart [15]–[17], [33], [34]. Hence, the administration of MSC-derived growth factors has been proposed as a novel therapy to treat ischemic heart disease, avoiding many practical and technical issues of cell therapy [20]. To this end, we previously reported that overexpression of GATA-4 in MSC not only upregulates the expression of these growth factors (e.g., VEGF and IGF-1) [30], but we now add that GATA-4 also regulates miRs in MSC and in MVs released from MSC.

Single-stranded miR usually binds to specific mRNA through sequences which are imperfectly complementary to the target mRNA. We have confirmed that miR-221/222 expression in MSC is higher than in CM and that GATA-4 upregulates miR-221 in MSC. Here we have observed that overexpression of miR-221 in MSC significantly reduces CM apoptosis. miR-221 is known to regulate the expression of Bcl-2 family that plays an important role in cell survival. Furthermore, overexpression of miR-221/222 increases cell survival, which is associated with the regulation of PUMA expression [24], [25]. PUMA, a pro-apoptotic BH3-only member of the Bcl-2 protein family, can neutralize anti-apoptotic proteins or activate pro-apoptotic proteins [26]. PUMA induces partial unfolding of BCL-xL, which disrupts interactions between cytosolic p53 and Bcl-xL to release p53 to initiate apoptosis [35]. We have shown here that the PUMA protein level in MSC is significantly less than that expressed in CM. Moreover, CM co-cultured with MSC have significantly lower PUMA levels, a result that is enhanced by MSC overexpressing miR-221. We have also compared the levels of miR221/222 and PUMA in CM which were co-cultured with MSC^GATA-4^ to that co-cultured with MSC^Null^. We found that miR-221 was higher (1.35 fold) and PUMA protein was lower in CM co-cultured with MSC^GATA-4^ than that co-cultured with MSC^Null^, but without significant difference. Our ongoing study shows that the benefit effect of cardioprotection of MSC^GATA-4^ is enhanced by a coordinated effect of several miRs which are regulated by GATA-4.

Normal and upregulated amounts of miRs can be released from MSC in MVs to act on neighboring cells. MVs are extracellular vesicles produced constitutively by most cell types and can be harvested from CdM [21], [22]. MVs not only contain numerous proteins and lipids, but they also contain mRNAs and regulatory miRs [36]–[38]. Interestingly, mRNA and miR molecules are preferentially enriched within MVs by a yet to be described process. It has been reported that the MVs obtained from cardiac progenitor cells enriched in miR-451 might be a promising cellular cardioprotection strategy [37]. MVs have been recently described as new mediators of cell-to-cell communication that may reprogram target cells through the radiational transfer of proteins, functional mRNAs, and miRs [36], [38]. We demonstrate here the transfer of bioactive miR carried by MVs from MSC into CM. We documented the CM internalization process of MVs using a Time-Lapse Imaging System. This system facilitates a broad array of long-term time lapse experiments with environmental controls during phase and fluorescence imaging of high resolution quality. Our studies indicate that CM rather quickly internalize MVs added to the cell culture system.

In this study, we not only investigated cardioprotection conveyed by CdM derived from cultures of MSC^GATA-4^, but also observed the translocation of miR-221 in MVs from MSC into CM, with the consequent reduction of PUMA in CM. Moreover, we confirmed that GATA-4 can increase miR-221 expression in MSC. Our studies suggest that MSC^GATA-4^ can deliver increased amounts of miRs within MVs to act on adjacent cells and to regulate target genes or protein expression within these cells.
